# An endocannabinoid catabolic enzyme FAAH and its paralogs in an early land plant reveal evolutionary and functional relationship with eukaryotic orthologs

**DOI:** 10.1038/s41598-020-59948-7

**Published:** 2020-02-20

**Authors:** Imdadul Haq, Aruna Kilaru

**Affiliations:** Department of Biological Sciences and Biomedical Sciences, East Tennessee State University, Johnson City, TN 37614 USA

**Keywords:** Enzymes, Plant sciences

## Abstract

Endocannabinoids were known to exist only among Animalia but recent report of their occurrence in early land plants prompted us to study its function and metabolism. In mammals, anandamide, as an endocannabinoid ligand, mediates several neurological and physiological processes, which are terminated by fatty acid amide hydrolase (FAAH). We identified nine orthologs of FAAH in the moss *Physcomitrella patens* (PpFAAH1 to PpFAAH9) with amidase signature and catalytic triad. The optimal amidase activity for PpFAAH1 was at 37 °C and pH 8.0, with higher specificity to anandamide. Further, the phylogeny and predicted structural analyses of the nine paralogs revealed that PpFAAH1 to PpFAAH4 were closely related to plant FAAH while PpFAAH6 to PpFAAH9 were to the rat FAAH, categorized based on the membrane binding cap, membrane access channel and substrate binding pocket. We also identified that a true ‘dynamic paddle’ that is responsible for tighter regulation of FAAH is recent in vertebrates and absent or not fully emerged in plants and non-vertebrates. These data reveal evolutionary and functional relationship among eukaryotic FAAH orthologs and features that contribute to versatility and tighter regulation of FAAH. Future studies will utilize FAAH mutants of moss to elucidate the role of anandamide in early land plants.

## Introduction

Endocannabinoids such as anandamide belong to a class of lipid derivatives referred to as *N*-acylethalomines (NAEs). These fatty acid ethanolamides are found in wide range of eukaryotic organisms such as yeast, *Caenorhabditis elegans*, bivalve mollusk, mammals and also plants^1–4^. The fatty acid chain length of these NAEs can range from 12 C to 20 C and are either saturated or unsaturated^5^. Among these NAEs, only anandamide or *N*-arachidonylethanolamide (NAE 20:4) is known to serve as a ligand to cannabinoid binding receptors to activate endocannabinoid signaling^6^. In mammals, while endocannabinoids are key participants in neural signaling and other physiological events^7–11^, other NAEs such as NAE16:0 serve protective function in a receptor-independent manner^12^. Irrespective of their receptor binding capability, the NAE type, content, composition and functions are highly diverse among organisms, in a tissue-specific manner. In *C. elegans*, endocannabinoids, including anandamide are essential for mobilization of cholesterol from internal reserves^13^ and they affect life span^14^. In human, circulating endocannabinoids are positively correlated to visceral adipose tissue mass^15^, while in rat, anandamide induces overeating by activating cannabinoid receptor^16^. In higher plants such as *Arabidopsis* *thaliana*, only 12 C to 18 C NAEs occur and they mediate growth, development and biotic and abiotic stress responses^17–21^. Interestingly, the fatty acid and NAE composition of early land plants such as bryophytes is distinct from that of higher plants^1^. For example, in the moss *Physcomitrella patens* and other bryophytes there is abundance of arachidonic acid and its derivative anandamide, which are absent in vascular plants^1^. Preliminary studies showed that anandamide content is about 20% of the total NAEs in *P. patens* and at higher concentrations (>10 µM) is a negative inhibitor of growth. However, there is no clear understanding of why anandamide was absent in land plants, which evolved later than bryophytes. Furthermore, the mechanistic role of anandamide, the identity of a potential receptor and the associated signaling network in *P. patens* remains to be discovered^22^.

The enzyme fatty acid amide hydrolase (FAAH) is highly conserved among eukaryotes^23–26^, which terminates NAE functions, including anandamide resulting in inactivation of the endocannabinoid signaling^23,26–28^. Thus far, FAAH has been extensively studied in *C. elegans*, mammals and model plant *Arabidopsis* *thaliana*. The distinct feature that separates FAAH from other amidase family proteins is a lysine-serine-serine based catalytic motif within the ~130 amino acids long sequence referred to as amidase signature (AS)^29,30^. While only one FAAH protein and its encoding gene are characterized in rodents, two proteins encoded by *FAAH1* and *FAAH2* were characterized in human and in *arabidopsis*^31–33^. There is 20% identity between human FAAH1 and FAAH2 proteins and they differ in their tissue-specific expression, substrate specificity, and function; FAAH1 has higher specificity for NAE 20:4 while FAAH2 prefers monounsaturated NAE (NAE 18:1)^29^. The human FAAH1 received greater attention of researchers due to its ability to terminate endocannabinoid signaling, which affects a number of physiological conditions including but not limited to Crohn’s disease, obesity, gastrointestinal disorder, cardiovascular disease, depression and apathy symptoms^34–38^. In *arabidopsis*, FAAH influences seedling growth, root length, stomatal closure and abiotic and biotic stress responses^39–42^. In general, when *FAAH* was knocked-out, irrespective of the organism, NAE levels increased but most often an associated phenotype was not obvious. In contrast, overexpression of *FAAH* enhanced growth and development of arabidopsis seedlings but compromised their ability to respond to stressors and abscisic acid^39,41,43^.

For mechanistic understanding of FAAH, crystal structure of mammalian FAAH with different inhibitors^30,44–48^, and recently, a plant FAAH, *A*tFAAH have been resolved^26^. These crystal structures aided in understanding the primary, secondary, tertiary and quaternary structures of the protein, mechanism of the catalytic reaction and also the chemistry involved in the entry of the substrate and release of the end product, free fatty acid. The catalytic mechanism of FAAH is unique when compared to other AS family proteins^23,49^. Series of mutagenic studies of mammalian FAAH suggested that Lys142, Ser217 and Ser241 are essential catalytic residues; the crystal structure of rat FAAH (Protein Data Bank (PDB) ID: 1MT5) revealed that these catalytic residues compose a novel catalytic triad in the mammalian FAAH^23,30,50^.

The crystal structure of AtFAAH (PDB ID 6DII) opened a new avenue for understanding the details of structural mechanism of plant FAAH in general and in relation to other eukaryotic FAAH in terms of substrate accommodation and catabolism^26^. The sequence identity between AtFAAH and RtFAAH is 34% with identical arrangement of the catalytic triad. Also, both arabidopsis and mammalian FAAH can dimerize^26,30^ and share characteristic features such as the occurrence of a membrane binding cap (MBC), membrane access channel (MAC), acyl binding channel (ABC), cytoplasmic access channel and substrate binding pocket (SBP)^26,30^, while their secondary structure formation is different. For instance, in RtFAAH, although a transmembrane (TM) domain is predicted in the N-terminus region, its deletion indicated that FAAH still binds to the membrane through MBC in α18 and α19 helices forming helix turn helix motif (amino acids 404–438). This motif basically interrupts the AS fold and forms the hydrophobic plateau domain facilitating the integration of FAAH into one leaflet of the lipid bilayer^23,30^. On the membrane face, an access port defined by Arg486 and Asp403 facilitates the substrate entry to the active site^30^. In contrast, the N-terminus region of AtFAAH is longer compared to RtFAAH and yet lacks a TM domain and its MBC is located in α1 and α2 helices^26^. Having MBC in a different region in relation to AS did not affect AtFAAH function. The MBC is considered to be important for an easy and direct substrate entry from the hydrophobic membrane bilayer^23^. The MAC is amphipathic and accommodates the entry and movement of polar NAE head groups to the active site of FAAH^23,26,30^. The MAC in RtFAAH coordinates with ABC by a ‘dynamic paddle’ composed of Phe432 and Trp531^44,51^. The dynamic paddle is not conserved in AtFAAH and its MAC and ABC are predicted to be a one large channel^23,26,30^. The cytoplasmic access channel on the other hand, is proposed to release the products into cytosol after catabolism of the substrate^23^. The SBP in both AtFAAH and RtFAAH is mostly hydrophobic but in AtFAAH, there are a number of hydrophilic residues making it more polar, which perhaps allows for accommodation of diverse substrates with or without the polar functional group^26^. Additionally, in AtFAAH, a distinct ‘squeeze and lock’ mechanism was proposed for ligand binding and release, which is absent in RtFAAH^26^. Studies thus far indicate some key similarities and differences between plant and animal FAAH, with regards to their structure, mechanism and substrate specificity.

Knowing the structural details of mammalian FAAH has helped a great deal in generating targeted therapeutics^52–54^. Further understanding of a distant plant FAAH, which might have evolved around 500 million years ago is expected to provide evolutionary and functional insights. Bryophytes are the first group of plants that successfully made transition from water to land and are naturally resilient to various stressors^55^. Interestingly, the lipid composition of these early land plants, including *P. patens* is distinct from that of higher plants^56,57^. Specifically, the identification of anandamide, along with other NAEs and its influence on the development prompted us to gain functional insights into the endocannabinoid catabolism and signaling in the moss. In this study, we not only identified a functional FAAH in *P. patens* but also predicted the relationship of its paralogs with other eukaryotic orthologs.

## Results and Discussion

### Putative moss FAAH with highest identity to its eukaryotic orthologs is an amidase

**Figure 1:**
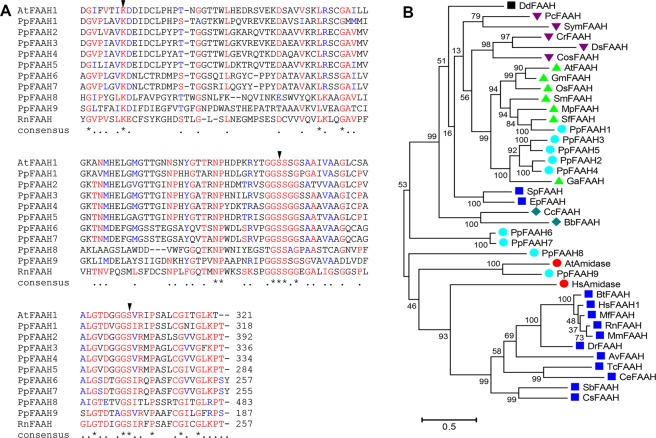
Alignment and phylogenetic analysis of FAAH. (**A**) Characteristic amidase signature (AS) of AtFAAH and RtFAAH were compared with nine FAAH candidates (PpFAAH1 to PpFAAH9) of moss. Arrows indicate the conserved catalytic triad of lysine-serine-serine. Numbers at the end of the sequences represents the last amino acid position of the AS. Alignment of full-length sequences is shown in Fig. S2. The symbols: asterisk, dot and gap for the consensus sequence indicate identical, same class and different class of residues at the same position, respectively. Red, green, and black colors also represent the same order of consensus symbols in terms of conserved residues. (**B**) Phylogenetic analysis of PpFAAH in relation to other eukaryotic orthologs (Table S3). Numbers indicate bootstrap values obtained from 500 replicates using the maximum likelihood method. The scale bar represents 0.5 amino acid substitutions per site.

To identify potential FAAH candidates with the ability to hydrolyze anandamide and other NAEs in the moss, *P. patens* database (v3.3) in Phytozome 12 was searched for homologs of rat, human and *arabidopsis* FAAH1. Nine moss proteins with high similarity to RtFAAH and AtFAAH were identified, which were considered putative and based on their order of homology and sequence identity were named chronologically, PpFAAH1 to PpFAAH9 (Table S1). The percentage identity of moss FAAH paralogs with AtFAAH, as generated by pairwise alignment ranged from 26% to 47% while with mammalian FAAH it was 28% to 34%. To obtain a more accurate identity among the sequences, percent identity matrix was generated by multiple sequence alignment using CLUSTAWL (Table S2). These data show that while PpFAAH1 shared similar identity with PpFAAH2 to PpFAAH5 and AtFAAH, which ranged from 46–44%, its identity with PpFAAH6 to PpFAAH9 was less than 26%. With mammalian FAAH, all the nine PpFAAH paralogs shared 18–25% identity. Among FAAH paralogs, PpFAAH2 to PpFAAH5 shared higher identity with each other than with the remaining PpFAAH; and while PpFAAH6 and PpFAAH7 shared highest similarity (85.6%) with each other, both PpFAAH8 and PpFAAH9 remained relatively distant from all other paralogs with <24% identity (Table S2). Interestingly, despite the differences in identity and the position of the AS region among these putative FAAH paralogs, the number of amino acids residues that make up the AS region remained between 122 to 124. All the nine paralogs not only retained the highly conserved AS sequence but also preserved the lysine-serine-serine catalytic triad (Fig. 1A). These nine FAAH paralogs of moss also showed diversity in their phylogenetic relationship to other eukaryotic FAAH (Fig. 1B). Together, these data suggest possibility for a shared functional relationship among these paralogs with some variations. First, to determine if these nine proteins are indeed paralogs of FAAH, we carried out biochemical characterization of putative PpFAAH1, including cloning, heterologous expression and purification, and radiolabeled enzyme assays (Fig. S1).

**Figure 2:**
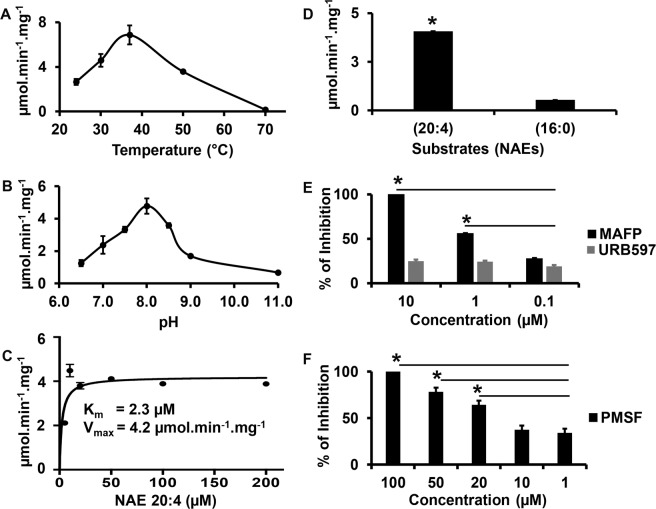
Amidohydrolase activity of PpFAAH1. Amidase activity at varying (**A**) temperature and (**B**) pH. (**C**) Saturation kinetics of PpFAAH1 with anandamide (NAE 20:4) as substrate; Km and Vmax values were calculated using Prism GraphPad. (**D**) Activity of PpFAAH1 with substrates anandamide (NAE 20:4; polyunsaturated) and palmitoylethanolamide (NAE 16:0; saturated). (**E**) and (**F**) The percentage of inhibition PpFAAH1 activity against anandamide by inhibitors - methyl arachidonyl fluorophosphonate (MAFP), [3-(3- carbamoylphenyl) phenyl] *N*-cyclohexyl carbamate (URB597) and phenylmethane sulfonyl fluoride (PMSF). Data represents mean values with standard deviation of three biological replicates. Statistical analysis (t-test) was performed using Prism GraphPad 8.0. The asterisk (*) sign and line (-) on top of the bar graphs represent significant difference relative to control without inhibitor (**D**–**F**).

Purified PpFAAH1 showed the ability to hydrolyze anandamide (Fig. S1) with an optimal activity at 37 °C and pH 8.0 (Fig. 2A,B), which was interestingly similar to that of HsFAAH2^29^. In contrast, both mammalian (HsFAAH1, RtFAAH) and Arabidopsis FAAH showed optimal activity at pH 9.0 but the temperature was 37 °C^29^ and 30 °C^24^, respectively. Additionally, at least 50% of amidase activity of PpFAAH1 was noted for a wide range of temperature (~ 25 to 50 °C) and pH (7.0 to 9.0), suggesting its adaptability to varying conditions. Furthermore, PpFAAH1 displayed typical Michaelis-Menten kinetics with a saturation curve (Fig. 2C). The kinetic parameters, Km and Vmax for PpFAAH1 with anandamide as a substrate were determined to be 2.3 μM and 4.2 μmol.min^−1^ mg^−1^, respectively (Fig. 2C), and with catalytic efficiency (Kcat) of 1.4 S^−1^ and specificity constant (Kcat/Km) of 0.61 μM^−1^S^−1^. In case of AtFAAH with NAE 20:4 as substrate, Km and Vmax of AtFAAH1 are 13.6 μM. and 0.29 μmol.min^−1^.mg^−1^, respectively with Kcat 0.33 S^−1^ and Kcat/Km of 0.024 μM^−1^S^−1^^25^. These data suggest that the catalytic efficiency and specificity of PpFAAH1 towards anandamide was more than four- and 25-fold higher than that of AtFAAH, respectively. Kinetic data for Hs/RtFAAH from various sources differed due the nature of samples and expression systems used and thus was not used for comparison here. Additionally, PpFAAH1 showed higher preference for polyunsaturated substrate (NAE 20:4) relative to a saturated NAE (NAE 16:0); the activity against NAE 16:0 was very low to determine its kinetic parameters (Fig. 2D). Interestingly, human FAAH1 also showed preference for polyunsaturated NAEs (NAE 20:4) while FAAH2 preferred monounsaturated NAEs (NAE18:1)^29^. Considering that there are nine potential FAAH candidates in *P. patens*, it would be of significance to identify if all the nine paralogs have amidase activity. Multiple FAAH, functional at different optimal conditions and diverse specificity towards NAEs and/or other substrates would allow for redundancy and flexibility in function in early land plants under varying environmental and physiological conditions.

### The conserved catalytic triad in FAAH orthologs is also responsible for PpFAAH1 activity

Specific endocannabinoid signaling inhibitors such as methyl arachidonyl fluorophosphonate (MAFP) and methyl α-linolenyl fluorophosphonate (MLnFP) interact with the catalytic triad of FAAH to inhibit its amidase activity. Among such analogs, PpFAAH1 activity was greatly affected by MAFP, which was potent at a concentration of 10 nM (Fig. 2E). Our subsequent *in silico* analyses showed how the polar head group of MAFP or MLnFP could make covalent bonds with the catalytic residues of PpFAAH1 to inhibit the hydrolysis of NAEs or fatty acid primary amides. Another well-known FAAH inhibitor, [3-(3- carbamoylphenyl) phenyl] *N*-cyclohexyl carbamate (URB597), which has a metabiphenylamide leaving group, a cyclohexyl moiety, and a carbamate reactive group showed an IC_50_ of 4.6 nM for mammalian FAAH^58^; in case of PpFAAH1, only 20% inhibition was achieved with 10 nM concentration (Fig. 2E). Structure analysis of URB597 bound Hs/RtFAAH revealed how a water molecule is likely involved with a different active site to inactivate enzyme by substrate diacylation^44^. Low inhibition of PpFAAH1 by URB597 indicates the possibility for different structural properties relative to Hs/RtFAAH. A serine protease, phenylmethanesulfonyl fluoride (PMSF) was also used to test the inhibition of PpFAAH1 as serine contributes to two residues in the catalytic triad. A 100% inhibition was accomplished with 100 μM concentration of PMSF (Fig. 2F). Although, similar concentration range of MAFP, PMSF and URB597 completely inhibited the activity of human^45,48^ or arabidopsis FAAH^25^, only MAFP and PMSF but not URB597 inactivated PpFAAH1 at a similar concentration. Inhibition by PMSF confirms that PpFAAH1 is also a serine hydrolase; inhibition by an anandamide analog, MAFP indicates the specificity of PpFAAH1 towards NAE 20:4; URB597 perhaps lacks appropriate structural interaction with its three benzene rings to successfully inhibit the activity of PpFAAH1. Nevertheless, these data unequivocally reveal that PpFAAH1 is indeed a hydrolase with ability to catabolize anandamide.

### Potential paralogs of moss FAAH1 reveal association with plant and animal FAAH

Considering the early evolution of mosses, relative to mammals or higher plants we attempted to understand the evolutionary relationship of the nine FAAH paralogs with other eukaryotic orthologs. To this extent, putative or known FAAH from 28 different species representing wide range of phyla from the five eukaryotic Kingdoms Protozoa, Chromista, Fungi, Animalia and Plantae were analyzed (Table S3)^59^ using Maximum likelihood method. The unrooted phylogenetic tree suggests an independent evolution of various FAAH orthologs, possibly from a common ancestral amidase protein that evolved much earlier to FAAH (Fig. 1B). The most ancestral FAAH to be characterized was that of *Dictyostelium*, a protozoan and was separated from the FAAH of Chromista, Fungi, Plantae, and two Cnidarians from Animalia, by a common ancestor (Fig. 1B). Most of the Animalia FAAH likely diversified from the orthologs of an amidase to form an independent clade. Separation of the cnidarian FAAH with the non-Animalia clade suggests that they evolved earlier and separately from the FAAH in higher phyla of Animalia. Interestingly, PpFAAH6 and PpFAAH7, which reflect duplication, AtAmidase and PpFAAH9, and PpFAAH8 show early and independent divergence from an ancestral protein. Also, not surprisingly, paralogs of PpFAAH1 to 5 were closest to orthologs from other bryophytes *Marchantia* (Mp) and *Sphagnum* (Sf)), followed by *Selaginella* (Sm), and then late vascular plants such as arabidopsis, rice (Os) and soybean (Gm). It appears that an early duplication of plant clade separated PpFAAH1 with other plant FAAH, which underwent a further duplication that separated the bryophyte and lycophyte FAAH from angiosperms. The second plant clade included PpFAAH2 to PpFAAH5 and FAAH from cotton. While PpFAAH1, which separated from the rest of its paralogs clearly showed an amidase activity, we speculate that the other paralogs perhaps have a related or redundant function. We carried out comprehensive predictive structural analyses to further assess the possible function of PpFAAH1 paralogs, which evolved at different time periods, in termination of endocannabinoid signaling.

### Paralogs of moss FAAH reveal unique and diverse features in relation to arabidopsis and mammalian FAAH

In comparison to AtFAAH and RtFAAH, paralogs of moss FAAH varied in sequence length, identity, predicted protein size (58 to 81 KDa), isoelectric point and number of TM domains (Tables S1 and S2). Nevertheless, higher identity was noted in the AS region of PpFAAH paralogs, AtFAAH and RtFAAH (Tables S1 and S2), than in their N- or C-termini (Fig. 1A, Fig. S2). Furthermore, in reference to the AS region, the N-terminus is extended in PpFAAH1 to PpFAAH4, like in AtFAAH and shortened in PpFAAH6 and PpFAAH7, as in RtFAAH. Unlike the other FAAH, PpFAAH8 has the longest N-terminus (Table S1, Fig. S2). Thus, the overall identity of N-terminus of PpFAAH1 to PpFAAH4 was higher with AtFAAH and PpFAAH6 and PpFAAH7 with RtFAAH. Both PpFAAH8 and PpFAAH9 showed less similarities because of their extended or shortened N-termini, respectively; PpFAAH5 on the other hand retained the conserved AS region but the remaining sequence lacked significant identity with either AtFAAH or RtFAAH (Fig. S1), and therefore, was not considered for comprehensive structural analysis. Although, the phylogenetic tree indicates PpFAAH5 and PpFAAH3 are likely a result of duplication, it is curious that they depart from each other significantly in predicted structural analyses. Additionally, PpFAAH8 includes fasciclin domain that attaches to the membrane with a lipid link^60^, and PpFAAH9 has a tetratricopeptide repeat domain, which serves as mediator of multiprotein complex. These interaction modules regulate diverse physiological processes in various organisms^61^, and thus suggests a unique role for PpFAAH8 and PpFAAH9 in moss. Using TMpred^62^, PpFAAH3, PpFAAH8 and PpFAAH9 were predicted to contain one TM domain from residues 39–61, 13–32, and 7–25, respectively in the N-terminus region. The probability for TM domain, however, was low at 0.2 for PpFAAH1 and none for the remaining paralogs. In contrast to AtFAAH1^26^, although RtFAAH possess a N-terminus TM^30^, its deletion did not interfere with the enzyme activity. In case of PpFAAH paralogs, the overall prediction analysis suggests that some share features of RtFAAH, while the others are more similar to that of AtFAAH. Such separation was also evident from the phylogenetic tree where only a few of the PpFAAH paralogs were closely related to AtFAAH (Fig. 1B). The occurrence of a number of potential amide hydrolases in the moss with features similar to both plant and animal FAAH suggests possible variability in terms of their function, substrate specificity and catabolism. Proteins with AS signature are typically involved in hydrolyzing a variety of substrates, in addition to NAEs such as, 2- arachidonoylglycerol, fatty acid primary amides and alkamides^6,23,31,63–65^. Such role for PpFAAH paralogs are expeccted to affect diverse physiological processes including growth, development, and responses to stress in early land plants^17–21^.

### Predicted structural variability of PpFAAH suggests evolutionary plasticity

**Figure 3:**
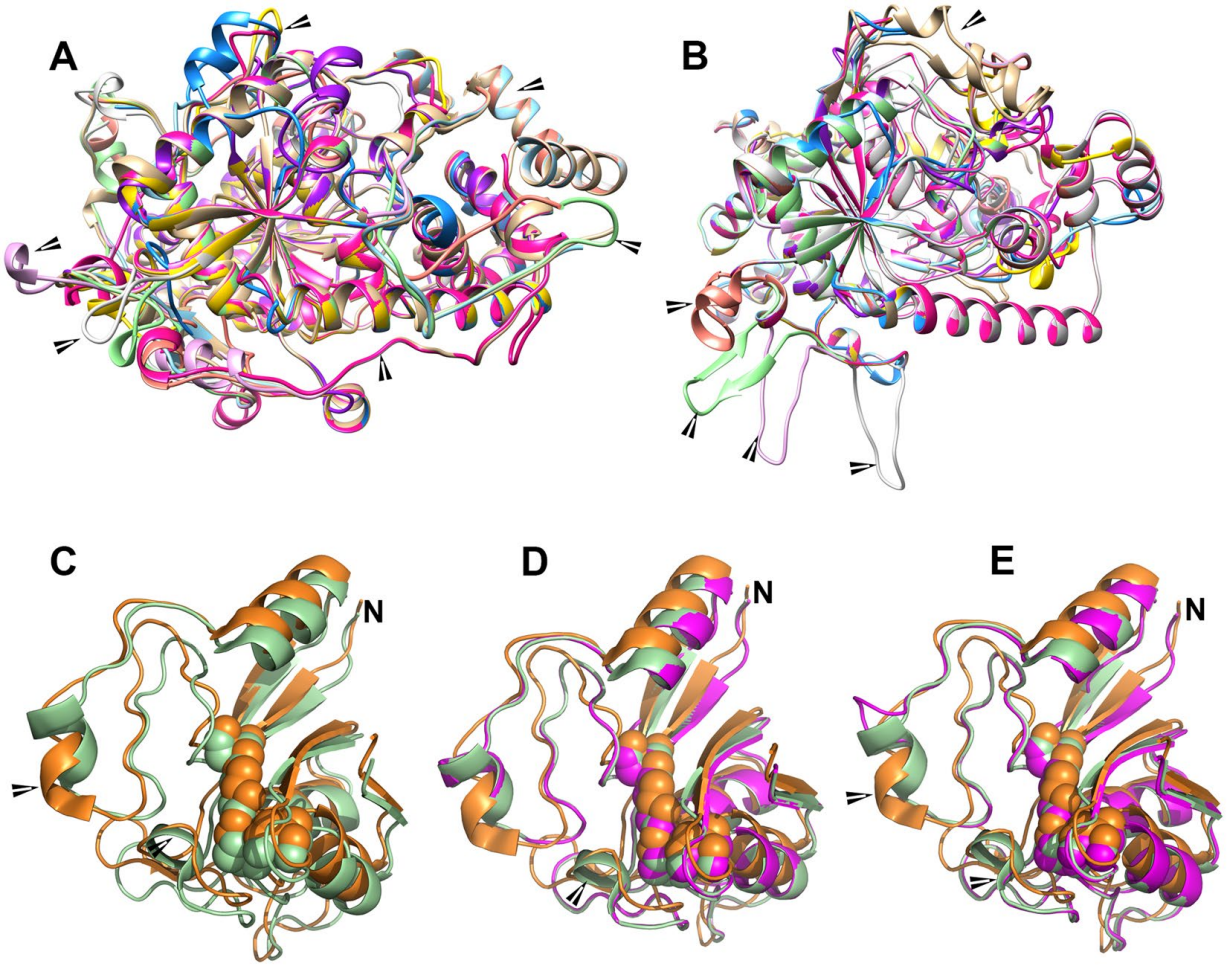
Comparison of predicted secondary structures of nine PpFAAH paralogs. An overlay of predicted secondary structures of nine PpFAAH with (**A**) AtFAAH (PDB ID: 6DII) and (**B**) RtFAAH (PDB ID: 1MT5) as templates. Structures were generated using Chimera 5 software. The comparisons predict that the secondary structures with both templates were similar with alpha-helices and loops surrounding the beta sheets core. Arrows point the differences on the aligned structure; Comparison of the amidase signature (AS) regions of (**C**) AtFAAH (green) and RtFAAH (orange) with arrow pointing to an additional α-helix in AtFAAH, and the two templates compared with (**D**) PpFAAH1 (purple) and (**E**) PpFAAH9. Arrows show the differences in the AS core. PpFAAH9 was predicted to make one less α- helix in the AS region compared to PpFAAH1. Catalytic triad is shown as sphere and N indicates the N-terminus of the AS region. Structural prediction and comparison for PpFAAH2 to PpFAAH8 is presented in Fig. S3.

Since protein structure is functionally more conserved than sequence similarity, the nature of secondary and tertiary structures of PpFAAH were also evaluated. The secondary structures were generated using PhyRe2.0 Protein Folding Recognition Server^66^ and with both AtFAAH (PDB ID: 6DII) and RtFAAH (PDB ID: 1MT5) as templates. The confidence of generating predicted secondary structures for all the nine PpFAAH paralogs with either template was 100%. The percentage coverage of the residues, however, varied with each comparison depending on the identity between template and the query (PpFAAH). For instance, when 6DII was used as a template, the sequence coverage of PpFAAH1 was 99.2% while it was only 72% with 1MT5 template (Table S4). Similarly, with 6DII template, the amino acid coverage for PpFAAH2 to PpFAAH6 was above 90%, while PpFAAH8 had the least with only 57% (Table S4). The coverage reduced from 85% to 57%, when 1MT5 template was used; interestingly, PpFAAH7 to PpFAAH9 showed similar coverage with both the templates. The secondary structure of AtFAAH1 contained 23 α-helices and 17 β-sheets where as RtFAAH has 22 α-helices and 11 β-sheets^26,30^. The number of α-helices in PpFAAH paralogs ranged from 25 to 16 and 8–10 β-sheets, when modeled with AtFAAH; this number varied in relation to RtFAAH (Table S4). Furthermore, the tertiary structure of PpFAAH was similar to that of AtFAAH and RtFAAH where the core is composed of a number of β-sheets, which are surrounded by α-helices and loops^26,30^. Since the length of the N- and C-termini of PpFAAH paralogs varied, the number of α-helices and loops surrounding the β-sheets also varied accordingly (Figs. 3A,B). In general, predicted secondary and tertiary structures of PpFAAH paralogs were less identical with each other relative to their comparison with AtFAAH and RtFAAH.

In addition to the highly conserved AS region, FAAH proteins also have characteristic features such as the MBC, ABC and MAC, which differed between AtFAAH and RtFAAH. The AS region of AtFAAH and RtFAAH makes four β-sheets but differ in the number of α-helices, which are five and four, respectively (Fig. 3C). The additional α-helix in AtFAAH is made by His252, Glu153, Leu154, Gly155 and Met256, whereas in RtFAAH, those residues are not conserved and instead make a loop. Similarly, the arrangement of α-helices, β-sheets and turns and loops of AS region of PpFAAH paralogs were almost identical to At/RtFAAH, even though the sequence identity between them ranged between 26% to 47% (Fig. 3C–E and S3). Additionally, like RtFAAH, the AS region of PpFAAH1 to PpFAAH5 also lacked the additional α-helix observed in AtFAAH (Fig. 3D, S3A–D). In contrast, PpFAAH6 to PpFAAH9 make one less α-helix in their AS region compared to other PpFAAH (Fig. 3E, S3E–G) and two less α-helices, relative to AtFAAH. Furthermore, although the position of the characteristic catalytic triad Lys-Ser-Ser in the primary sequence varied among all the paralogs of PpFAAH (Table S3), depending on the size of the protein they were spaced out with same distance within the sequence and closely match the fold of either AtFAAH or RtFAAH (Fig. 3 and S3). For instance, the distance between the nitrogen atom of lysine and oxygen of serine is ~2.6 Å and nitrogen of serine to oxygen of serine is ~3.0 Å. The catalytic triad position of PpFAAH1 (K202, S278, S302) is very similar to AtFAAH (K205, S281, S305)^26^ but PpFAAH6 (K139, S215, S239) and PpFAAH7 (K142, S217, S241) have positioning similar to that of RtFAAH (K142, S217, S241)^30^.

Furthermore, in comparing the features a dimer, both AtFAAH and RtFAAH show a symmetric pattern by which the orientation of the protein subunit align in a way that MBC and MAC of each subunit are placed on the same face of the dimer^23,26,42^. Several amino acid residues that interact by hydrogen bond and Van der Waals interactions for dimerization were also identified; in AtFAAH, four residues are present in N-terminus and two in α17 and α20^26^. Structural comparison of PpFAAH1 to PpFAAH4 with AtFAAH revealed that either identical or same class of residues are present in the same regions (Table S5), suggesting the potential of PpFAAH to function as a homodimer. However, with a number of FAAH candidates in *P. patens*, forming heteromers to attain functional diversity is also a possibility. Overall, PpFAAH1 to PpFAAH5 have more structural similarities with AtFAAH, while PpFAAH6 to PpFAAH9 showed better match with RtFAAH, which agrees with the way they clustered in phylogenetic tree and thus suggesting a possible functional similarity. The subtle structural variabilities among PpFAAH paralogs also points to likely flexibility in substrate utilization that is perhaps associated with plasticity in evolutionary adaptation of mosses to environmental variation.

### The membrane binding cap in PpFAAH shares similarities with mammalian and plant FAAH

**Figure 4:**
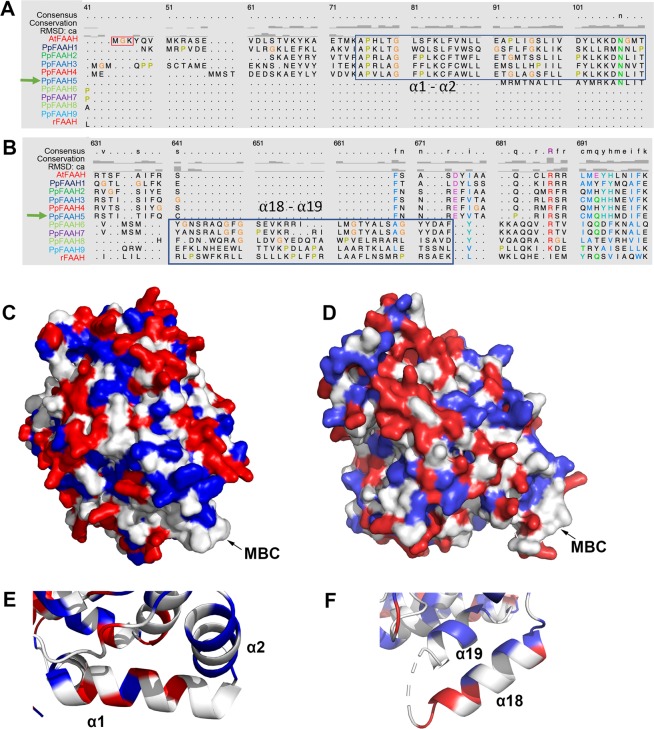
The membrane binding cap (MBC) properties of PpFAAH. Sequence alignment of PpFAAH with AtFAAH and RtFAAH using Chimera 5 to identify potential MBC in (**A**) N-terminus and (**B**) C-terminus. Boxes indicate conserved residues of PpFAAH paralogs either with AtFAAH to form the α1 and α2 or with RtFAAH to form α18 and α19, for MBC; Spatial surface structures showing MBC of (**C**) PpFAAH1, (**D**) PpFAAH6, and the close up their respective alpha helices in (**E**) and (**F**). White, red and blue colors represent the hydrophobic, charged and polar residues, respectively.

In AtFAAH, MBC is formed in the α1 and α2 helices of the N-terminus, of which 21/34 residues are hydrophobic and are arranged like teeth on a comb^26^. In RtFAAH, since the N-terminus is shorter and has a TM domain, MBC is formed in α18 and α19 helices of C-terminus with 23/34 hydrophobic amino acid residues^30^. Like AtFAAH, PpFAAH1 to PpFAAH4 have MBC in their relatively longer N-terminus (Fig. 4A,C,E and 3C), while PpFAAH6 and PpFAAH7, like RtFAAH with shorter N-terminus have their MBC in the C-terminus (Fig. 4B,D,F and 3D). Both PpFAAH8 and PpFAAH9, with their longest and shortest N-terminus, respectively also have their MBC in the C-terminus. Similar to AtFAAH, the MBC in both PpFAAH1 and PpFAAH2 is predicted in α1 and α2 helices with 24/41 and 19/37 hydrophobic residues arranged from L21-P61 and A94-L127, respectively (Fig. 4A, Table S6), and are arranged like teeth on a comb. For PpFAAH3 and PpFAAH4, a TM integrated MBC is predicted in the α1 and α2 helices in N-terminus with higher ratio of hydrophobic residues19/23 and 24/31, respectively (Table S6). The N-termini of PpFAAH6 and PpFAAH7 do not align with that of AtFAAH and are predicted to have MBC in the α18 and α19 helices of the C-terminus, but with a high ratio of hydrophobic residues that align with RtFAAH (Fig. 4B, Table S6). Although the coverage of predicted secondary structure for PpFAAH8 and PpFAAH9 was relatively low, by comparing them to AtFAAH and RtFAAH, even with the limited coverage, it is predicted that C-terminus could make the MBC. Residues A602-V616 in α12 and V258-L271 in α9 have the potential to make MBC for PpFAAH8 and PpFAAH9, respectively. Predicted secondary structure of PpFAAH5, on the other hand, did not reveal any hydrophobic plateau in either termini, and thus the MBC region was not predicted (Table S6). Wherever the MBC was predicted for PpFAAH, irrespective of the termini, they could be membrane integrated or arranged as teeth on a comb.

### The moss FAAH paralogs split to share similarities with AtFAAH and RtFAAH for substrate interaction

In both AtFAAH and RtFAAH, to access substrates such as NAE from the membrane, the MAC starts at the edge of the MBC in α1 and α2, and α18 and α19 helices, respectively^26,30^. The entrance of ligand binding pocket for AtFAAH is constituted with a number of hydrophobic (A27, P28, L30, P38, I51, and L55) and two charged (K26 and D58) residues. Similar to AtFAAH, the entrance of the ligand binding pocket for PpFAAH1-PpFAAH4 are also found in the α1 and α2 helices and have all the hydrophobic residues conserved, except PpFAAH4 has leucine instead of isoleucine (Table S7, Fig. S4). Among the two charged residues, both are conserved in PpFAAH4, but valine is replaced aspartic acid in PpFAAH3 and lysine with arginine in PpFAAH2; there are no charged residues in PpFAAH1 and instead two hydrophobic residues (isoleucine and methionine) are present in the same structural position (Table S7, Fig. S4). Nevertheless, PpFAAH1 to PpFAAH4 made the same predicted secondary structure as AtFAAH (Fig. S5A).

The SBP in both AtFAAH and RtFAAH are also composed of mostly hydrophobic and few hydrophilic residues^26,30^. The acyl chain of the substrate interacts with hydrophobic residues by Van der Waals interaction, whereas hydrophilic residues in the SBP helps to move the polar head group of the substrate deeper into the pocket towards the catalytic triad^23^. Analyzing the SBP of PpFAAH1 to PpFAAH4 revealed that the hydrophobic residues that form the SBP in AtFAAH (M25, A27, L55, M61, G255, M256, G257, V442, I445, I475, F476, F479, I532 and M539) are either conserved or replaced with another hydrophobic residue (Table S8). The hydrophilic residues (N59, T258, H441, S472, T535, and T536) of AtFAAH make its SBP relatively more polar than that of RtFAAH. Out of the six hydrophilic residues in AtFAAH, three of them (H441, S472 and T535) are also conserved among other higher plants^26^; however, that is not the case for PpFAAH1 to PpFAAH4. While N59, T258 and H441 are conserved T535 and T536 are replaced by hydrophobic residues (Gly, Val or Ala) in all four PpFAAH; S472 was replaced with the same class threonine for PpFAAH1, PpFAAH3 and PpFAAH4 (Table S8). These reduced number of hydrophilic residues suggest that the SBP of PpFAAH1 to PpFAAH4 is less polar than AtFAAH but remains more polar than RtFAAH.

In case of PpFAAH6 to PpFAAH9, since their MBC is predicted in the C-terminus, the entrance of the ligand binding pocket, MAC and SBP depart from AtFAAH, and instead they are similar with RtFAAH. Structural alignment of PpFAAH6 to PpFAAH9 with RtFAAH revealed that the positioning of the residues that contribute to MAC and SBP are mostly conserved with a few exceptions where they are either replaced with same or different class of residues, and thus retain the structural identity (Table S5, Fig. S5B). Specifically, of the 29 residues of RtFAAH SBP, nine are substituted with different class of residues; six of nine were hydrophobic and three hydrophilic. In SBP of PpFAAH6 and PpFAAH7, six were hydrophilic and three were hydrophobic making them more polar than RtFAAH and thus flexible to accept substrates of that nature. As mentioned earlier, the coverage of the predicted secondary structure of PpFAAH8 is relatively low, which is reflected in the predicted SBP and their secondary structure arrangement (Table S9). In conclusion, the MAC and SBP for PpFAAH1 to PpFAAH4 and PpFAAH6 to PpFAAH9 are predicted to be similar to AtFAAH and RtFAAH, respectively.

**Figure 5:**
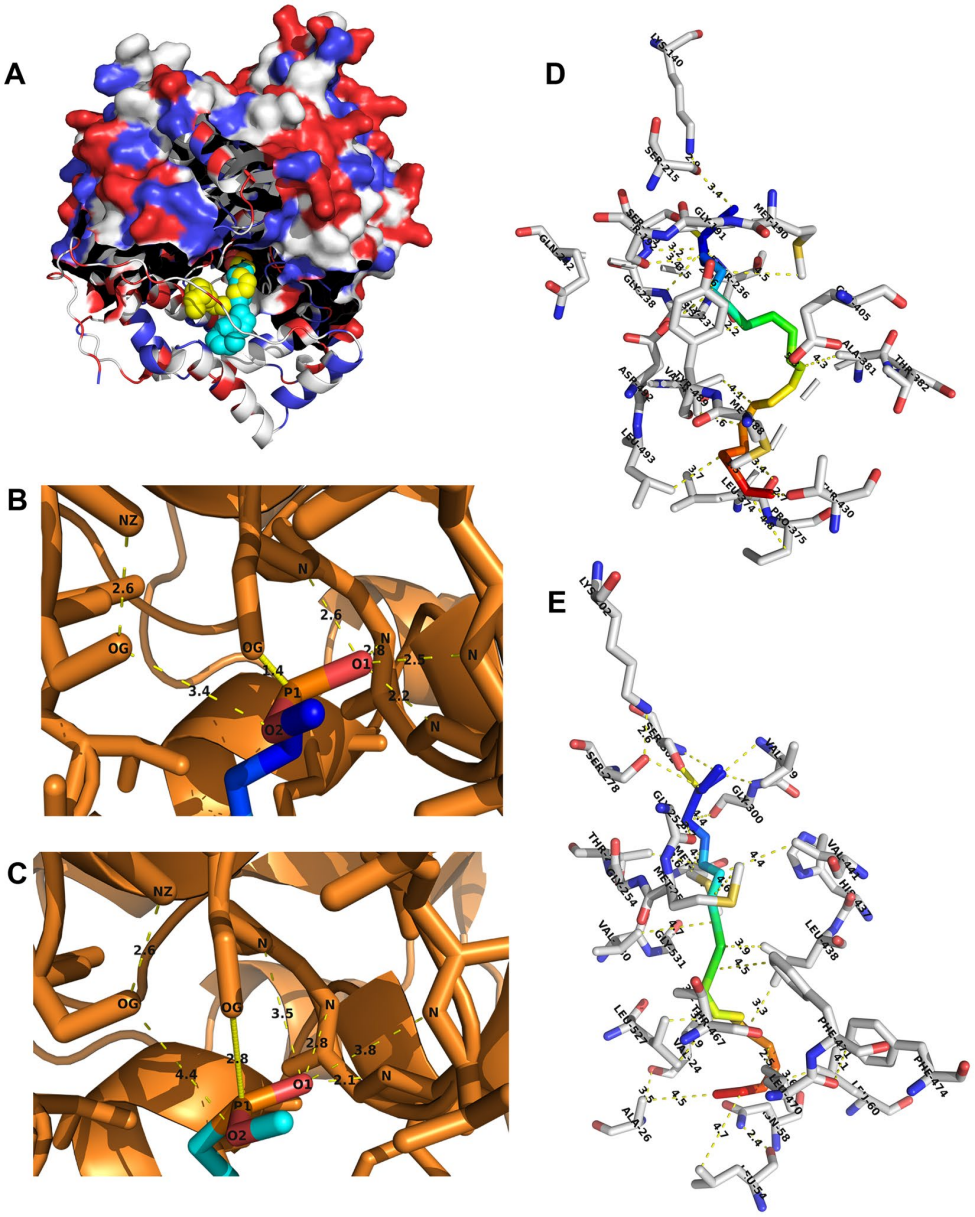
Substrate docking of PpFAAH1 and PpFAAH6. (**A**) The structure of PpFAAH1 along with docked substrate analogs, MAFP (yellow) and MLnFP (green) are presented. The PpFAAH1 structure is shown in a partial space-filling model with secondary structures as ribbon, interacting with substrate shown as sphere. (**B**) Polar interaction of MAFP head group with catalytic residues of PpFAAH1. (**C**) The polar interaction between MLnFP head group and catalytic residues of PpFAAH1. The distance (in angstrom) between the atoms were shown with yellow dotted lines. The nucleophilic attack on the phosphorus of substrate by Ser302 shown as solid yellow line. Atoms are labeled with atomic symbols (N, nitrogen; O, oxygen; P, phosphorus). (**D**) Substrate MAFP is shown in the substrate binding pocket of PpFAAH1 and (**E**) PpFAAH6. Van der Waals interaction between residues and acyl chain of MAFP (in rainbow color) are shown with dashed yellow lines. Amino acid residues are notated with their three-letter code and position while their carbon, oxygen and nitrogen are represented as gray, red and blue respectively.

Previously, crystal structures of AtFAAH and RtFAAH were generated using analogs MLnFP (analog of 18 C NAE) and MAFP (analog of anandamide), respectively. To identify potential substrate preference of PpFAAH, both the analogs were docked using Auto Dock Vina in PyRx software. Substrate docking analysis for PpFAAH paralogs revealed that substrates interact with residues in the predicted SBP *via* Van der Waals forces or polar-covalent bonds. Van der Waals interactions occur between hydrophilic acyl chain of the substrate and residues in the SBP. The polar-covalent interaction occurs between the polar head of the substrate and catalytic residues. Among the nine paralogs PpFAAH1 and PpFAAH6, were selected as the representatives that were predicted to be closer to AtFAAH and RtFAAH, respectively and analyzed (Fig. 5). The head group of both substrates are identical, but the tails or acyl chains are different in number of carbon and double bonds. Because of their structural differences in tails, entry and accommodation of the substrates in to the SBP of the enzyme are different as the residues of SBP that interact by Van der Waals force with each of the substrates vary. The polar interaction between the head group and catalytic residues are the same positional wise, however, the distance between the catalytic residues and the polar head groups of the substrates are different which determines how efficiently substrate will undergo a nucleophilic attack by the catalytic residue of S302 of PpFAAH1 (Fig. 5A). In contrast, PpFAAH6 docking revealed that the positioning of the substrate inside its secondary structure was different from that of AtFAAH and PpFAAH1 to PpFAAH4 but rather similar to RtFAAH (Fig. 5C, Table S9). Docking analysis of MLnFP or MAFP with PpFAAH1 showed that catalytic nucleophile Ser302 makes polar-covalent bond with phosphorus of the substrate, and oxygen atoms make polar covalent bonds with nitrogen atom of Ser278, Val299 and Gly300 (Figs. 5A,B). On the other hand, for PpFAAH6 substrate docking showed that catalytic nucleophile Ser239 makes polar bond with phosphorus of MAFP (Fig. 5C). In RtFAAH, Ser241 and in AtFAAH Ser305 form covalent bond with phosphorous of MAFP and MLnFP, respectively^26,30^ This analysis suggested that even though the path of MAC or SBP is different among rat, arabidopsis or moss FAAH, the catalytic mechanism remained identical.

### Fully conserved ‘dynamic paddle’ feature is likely limited to Phylum Chordata

**Figure 6:**
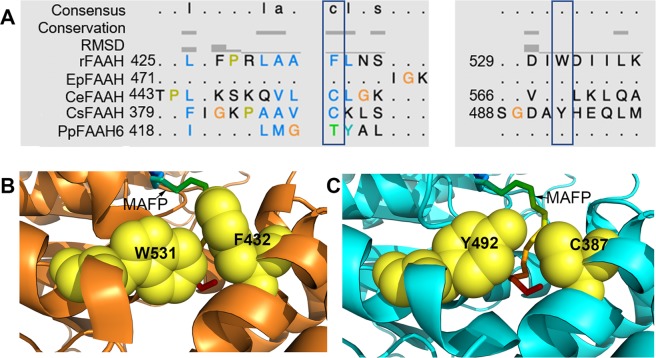
The dynamic paddle of FAAH. (**A**) Structural alignment of EpFAAH, CeFAAH, CsFAAH and PpFAAH6 with F432 and W531 regions of RtFAAH; boxes represent the position associated with dynamic paddle residues of RtFAAH. (**B**) Formation of dynamic paddle by W531 and F432 of RtFAAH with docked MAFP. (**C**) Formation of dynamic paddle like structure in CsFAAH with substituted residues of Y492 and C387 in place of W531 and F432, respectively. Substrate, MAFP is shown as a stick with rainbow color. Residues of dynamic paddle are shown as sphere and yellow in color. Formation of dynamic paddle in these five FAAH orthologs shown in the alignment are presented in Fig. S7.

Key difference of RtFAAH from that of PpFAAH and AtFAAH is the presence of ‘dynamic paddle’ residues, F432 and W531 in the C terminus of 18 to 21α helices that separate SBP/ABC and MAC. The ‘dynamic paddle’ of FAAH enzyme plays an essential role in terms of substrate selectivity and its accommodation from MAC to SBP/ABC pocket^51^. Double mutation of Phe432 and Trp531 to alanine in RtFAAH, along with microsecond-long molecular dynamic simulations study revealed the significance of these two residues in substrate selection and catabolic rate^51^. Change in specificity of substrate selection and the rate of catabolism by human FAAH are associated with a number of disorders, including but not limited to weight gain, energy balance, food intake, and anxiety control, etc^67–69^. Therefore, ‘dynamic paddle’ residues are considered crucial in tight regulation of substrate selection, entrance and its catabolism and thus, prevention of various diseases. To further understand the role and evolution of dynamic paddle, predicted FAAH structure of 28 organisms, representing select phyla of Kingdom Protozoa, Chromista, Fungi, Plantae and Animalia were analyzed. Unlike in RtFAAH and HsFAAH, none of the plant FAAH analyzed were predicted to make the α helices with W531 to constitute the ‘dynamic paddle’ (Fig. S6B). Among those examined, only PpFAAH6 to PpFAAH9 were predicted to make the α helix in the region where F432 of RtFAAH occurs, but phenylalanine was not conserved (Fig. S6A, S7). Among the FAAH orthologs that were analyzed, tryptophan and phenylalanine were only conserved in the Phylum Chordata; organisms analyzed were- human, rat, mouse, cow, rock chuck and zebrafish; both the residues were not conserved in any other phyla of the five Kingdoms examined (Fig. 6, S6A).

In Kingdom Animalia, among the 13 species that were tested for similarities in dynamic paddle structure, lower organisms like *Stylophora pistillata* and *Exaiptasia pallida* from Cnidaria Phylum did not make the predicted α helices in that region in which W531 of RtFAAH is present (Fig. 6, S6A). Nematodes such as *Toxocara canis* and *C. elegans*, however, made the α helices but tryptophan was not conserved. Among the Platyhelminthes, *Clonorchis sinensis* and *Schistosoma bovis* were also predicted to make α helices, but tryptophan was substituted with same class of aromatic amino acid, tyrosine (Figs. 6A,C, S6A, S7E). The occurrence of another residue in dynamic paddle, phenylalanine also showed the same trend in Kingdom Animalia. Organisms from Nematoda and higher Phyla, including Platyhelminthes and Arthropoda were predicted to make the α helices but the position aligned with F432 of RtFAAH was substituted by a different class of amino acid (Fig. 6A, S6A). These analyses indicate that dynamic paddle-like structure slowly evolved from lower to higher organisms. For example, among the organisms analyzed, Phylum Cnidaria was not predicted to make any of the α helices in which W531 and F432 are present. Phylum Nematoda could make the α helices corresponding to the region of F432 and W531 but without the two residues. Phylum Platyhelminthes was predicted to make the α helices associated but with substituted residues to Phe and Trp (Fig. 6, S6, S7). Moreover, when substrate docking (MAFP) on FAAH from respective organisms was observed, we detected that FAAH from *Clonorchis sinensis*, a Platyhelminth forms a structure similar to that of dynamic paddle but the space that accommodates substrate entry through MBC to the SBP is wider compared with RtFAAH (Fig. 6B, S7D,E); recent study suggested that this space is important for a tight regulation of substrate entry^51^. Interestingly, although the occurrence of endocannabinoids is widely reported among eukaryotes, the identification of endocannabinoid signaling system, including the cannabinoid-binding receptors are thus far limited to chordates. This observation led us to hypothesize that organisms in Animalia kingdom evolved dynamic paddle-like structure in FAAH, as complexity increased and tighter regulation of substrates became crucial, leading to the true dynamic-paddle. As such, it is likely that the complexity in regulation of the enzyme evolved as a necessity associated with functional implications of the signaling network. Among the nine PpFAAH, only PpFAAH6 to PpFAAH9 were predicted to make the α helices in the region corresponding to RtFAAH F432. This observation further justifies the versatility of FAAH in *P. patens*, which is an important transitional species in evolution.

## Conclusions

Structural variations of a highly conserved enzyme across diverse phyla are critical for understanding functional evolution. Crystal structures of mammalian and plant FAAH served as templates to predict and understand structural details of uncharacterized amidase family of proteins. In the moss, *P. patens*, it is evident that there is a functional FAAH that can not only metabolize anandamide but does so with higher specificity and catalytic efficiency of AtFAAH or RtFAAH. Additionally, ability of PpFAAH1 to retain more than 50% activity at a wide range of temperature and pH also suggests the adaptability of the enzyme to varying conditions. Such adaptations by enzymes are likely to rely on structural alterations that affect ligand binding and catalytic rate. The enzyme FAAH being a highly conserved protein across the eukaryotic lineage that evolved about 1.5 billion years ago, along with multiple paralogs of FAAH in moss provide a unique resource to further explore their successful functional and structural adaptability to shifting environmental conditions during evolution.

Our comprehensive and systematic *in silico* analyses of identification and evaluation of structural details of PpFAAHs also alluded to divergence of FAAH between the plant and animal lineages. While the AS region and catalytic triad appear to be universally conserved among the FAAH orthologs, variations in other key functional features such as the MBC, MAC, SBP, ABC and extended or shortened N or C termini evidently contributed to their structural and perhaps functional divergence. Of interest here is the clear splitting of moss FAAH paralogs into either *arabidopsis* or rat FAAH related, based on these additional key features. While the moss FAAH1 paralogs might in general possess the potential for amidase activity, as indicated by the interaction of their catalytic site with substrate analogs such as MAFP and MLnFP, their differentiation into two lineages provides us additional tools to evaluate the subtle variations that are predicted to affect specificity, catabolic rate, enzyme-ligand interaction and regulation and varying physiological conditions for optimal activity.

Recent studies have shown the significance of two gating residues Phe432 and Trp531 in forming the catalytic site of FAAH, which selectively accommodates anandamide and orients for efficient hydrolysis. Comparative analysis of dynamic paddle region among FAAH orthologs from various phyla, curiously revealed that the true dynamic paddle region with Phe and Trp residues was limited to orthologs from Chordates, which might be associated with the need for evolution of tighter regulation of the endocannabinoid signaling system. Like in mammals, because of the occurrence of anandamide in mosses, we expected PpFAAH paralogs to show a conserved dynamic paddle region. However, the absence of such region among PpFAAH paralogs suggests that they might be more versatile in their lipid selection for substrates. In mosses, the class of NAEs, in addition to anandamide include other saturated and unsaturated NAEs ranging from 16 C to 20 C. Thus, it is plausible that these paralogs are more attuned to accept these diverse range of substrates.

Our studies conclude that NAE-mediated functions in moss can be complex and diverse and various FAAH paralogs are likely to play a role in their hydrolysis. The structural similarities and dissimilarities identified among the various orthologs provides us basis to understand the diversity among them. Finally, the role of anandamide in mammals and its regulation by FAAH implicates that such a system in mosses is also likely to affect various physiological aspects and address how early land plants might deal with extreme biotic and abiotic stressors.

## Materials and Methods

### Identification and *in silico* analyses of FAAH homologs

Protein sequence of FAAH from human (NP_001432.2), rat (NP_077046) and arabidopsis (AT5G64440.1) were used as query sequences to search for homologs in *Physcomitrella patens* using *Phytozome 12 Physcomitrella patens v3.3* database. Best hits for putative PpFAAH (1–9) thus obtained were further used for multiple sequence alignment and to obtain percent matrix identity against rat and arabidopsis FAAH using Clustal Omega Multiple Sequence Alignment^70^. The MEGA7 software was used to generate phylogenetic tree using FAAH protein sequences from 29 different organisms (Table S3) representing five eukaryotic kingdoms. For this MEGA and Maximum Likelihood method based on Subtree-Pruning-Regrafting (SPR) algorithm with search label 1 was used to generate the phylogenetic tree. For the test of phylogeny, bootstrap method with 500 replicates was used; substitute type was amino acid; Gaps/Missing Data Treatment was used for partial deletion with 95% coverage cutoff^71^. A human amidase (NP_777572) and arabidopsis amidase (AT1G08980.1) sequences that were not FAAH served as outgroups.

### Cloning, expression, and purification of heterologous PpFAAH1

The moss *Physcomitrella patens* (ecotype Gransden 2004) was cultured on BCD media at 25 °C with light intensity of 17.45 W/m^2^ under 16-h light and 8-h dark cycle. Total RNA was extracted using plant mini RNA kit (Qiagen) and cDNA was synthesized using reverse transcriptase (Promega) following manufacturer’s protocol. Full length *PpFAAH1* was amplified using forward (5’-AAAAAGCAGGCTCGATGGCGCAAAATAAGATGCGAC-3') and reverse primers (5'-AGAAAGCTGGGTCTTACTTCAAGATGTTATAGAATG-3') including STOP codon. Amplified *PpFAAH1* was then cloned into pDEST15 vector following Gateway cloning protocol. For expression, *PpFAAH1* containing vector was then transformed into *E. coli* host BL21(DE3) cell line. The 1 mM IPTG induced cultures were incubated for 4 h at 37 °C and harvested cells were French pressed with lysis buffer (50 mM Tris-Cl pH 8.0, 100 mM NaCl, 1% Triton-100), and then purified by GST-tagged Spin Purification Column Kit (Thermo-Fisher). Purified PpFAAH1 was further concentrated by Amicon Ultra-15 (Ultracel −30K), quantified by using Nanodrop (ND-1000) and further confirmed by Western blot (Text S1).

### Fatty acid amide hydrolase (FAAH) assays

For FAAH amidohydrolase assay, 3 μg of purified PpFAAH1 was used for all experiments. To determine optimal conditions, pH range from 6.5 to 11, and a temperature range of 24 °C to 70 °C were used. For kinetic assays, pH 8.0 and 37 °C were used as optimal conditions. To determine substrate specificity for PpFAAH1, [1-^14^C]NAE20:4 and [1-^14^C]NAE16:0 were used. While AtFAAH was used as positive control, GST protein and no enzyme were used as negative controls. Since optimal condition for AtFAAH activity is different^26^, kinetic comparisons were not made between AtFAAH and PpFAAH1 in this current study.

Amidohydrolase reaction assay and lipid extraction for product analysis were carried out as previously described^72^. Thin layer chromatography (TLC) plate (TLC Silica Gel 60, Millipore) was used to separate lipid soluble reaction products and a radiometric scanner (Eckert & Ziegler, AR2000) was used to quantify the FAAH activity.

For FAAH inhibition assays, the above protocol was followed except that the reaction mixture with enzyme was incubated with an inhibitor for 10 min prior to the addition of 100 μM substrate [1-^14^C]NAE20:4. Three inhibitors, PMSF, MAFP and URB597 were used in the assays, with varying concentrations (1 to 100 μM for PMSF; 0.1 to 10 nM for MAFP and URB597).

### Secondary structure prediction and molecular docking

Secondary structures of putative PpFAAH were generated using Phyre 2.0 Protein Folding Recognition Server^66^ and using both RtFAAH (PDB ID 1MT5) and AtFAAH (PDB ID 6DII) as templates. For FAAH from 27 different organisms (Table S3), secondary structures were generated using either RtFAAH or AtFAAH, depending on their respective Kingdom. The PDB format of secondary structure for PpFAAH were further analyzed using Chimera 5.0^73^ and/or PyMOL software. For quality control, the predicted secondary structures of RtFAAH and AtFAAH were also generated using the same resource, Phyre 2.0. For quality check, we have also used PDBeFold (PDB in Europe, Structure Similarity)^74^ by which several criteria of 3D structure between template and query were analyzed. We reported Q and root-mean standard deviation (RMSD) scores ranging from 0–1, where higher Q score and lower RMSD indicates better quality.

Crystal structures of AtFAAH (PDB ID 6DII) and RtFAAH (PDB ID 1MT5) were previously generated with inhibitors MLnFP^26^ and MAFP^30^, respectively. These structures, obtained from PDB database were used for substrate docking analyses of PpFAAH, and further understanding of plant and animal FAAH. The PDB format PpFAAH structures with substrates were docked using Auto Dock Vina of PyRx software^75^. Docked structures were then visualized using PyMOL.

## Supplementary information

Supplementary Information.
