# Supplementary material for: An endocannabinoid catabolic enzyme FAAH and its paralogs in an early land plant reveal evolutionary and functional relationship with eukaryotic orthologs
Source: Sci Rep. 2020 Feb 20;10:3115. doi: 10.1038/s41598-020-59948-7 (PMC7033180; doi:10.1038/s41598-020-59948-7)
Supplement: Supplementary file 1 — Supplementary Information. [file 41598_2020_59948_MOESM1_ESM.pdf]

Supplementary Information for

An endocannabinoid catabolic enzyme FAAH and its paralogs in an early land plant reveal evolutionary and functional relationship with eukaryotic orthologs

Imdadul Haq[1] and Aruna Kilaru[1#]

Aruna Kilaru
Email: kilaru@etsu.edu

This PDF file includes:

Supplementary text
Figures S1 to S7
Tables S1 to S9

Supplementary Information Text

Methods

Western blot. To confirm GST-tagged PpFAAH1 expression in *E. coli*, protein extract was first separated by SDS-PAGE (10%) and then transferred to polyvinylidene difluoride membrane, blocked with 1% milk and 3% BSA. The membrane blot was incubated overnight at 4°C with monoclonal anti-GST antibody (1:1000 dilution). The blot was then washed three times with phosphate-buffer saline (PBS) followed by 1X PBS with 3% Tween-20 (PBST), and PBS. Anti-mouse secondary antibody (1:3000 dilution) was added to the blot and incubated for one hour at room temperature. Blot was then washed sequentially with PBS, PBST and PBS, and was then subjected to enhanced chemiluminescent (ECL) HRP and AP substrates, and finally exposed on x-ray film (Fig. S1).

In silico analysis. To estimate the molecular weight and calculate the isoelectric point (pI), UniPort Knowledgebase (Swiss-Port or TrEMBL)[1] was used. The Clustal Omega Multiple Sequence Alignment online tool was used for multiple alignment and BoxShade (https://embnet.vital-it.ch/software/BOX_form.html)[2] was used to obtain print quality alignment file. For transmembrane domain analysis TMHMM2.0[3] and TMpred[4] were used.

References:

1. Gasteiger, E. *et al.* Protein Identification and Analysis Tools on the ExPASy Server. in *The Proteomics Protocols Handbook* 571–607 (Humana Press, 2005). doi:10.1385/1-59259-890-0:571
2. Sievers, F. *et al.* Fast, scalable generation of high-quality protein multiple sequence alignments using Clustal Omega. *Mol. Syst. Biol.* **7**, (2011).
3. Sonnhammer, E. L., von Heijne, G. & Krogh, A. A hidden Markov model for predicting transmembrane helices in protein sequences. *Proceedings. Int. Conf. Intell. Syst. Mol. Biol.* **6**, 175–82 (1998).
4. Ikeda, M., Arai, M., Okuno, T. & Shimizu, T. TMPDB: a database of experimentally-characterized transmembrane topologies. *Nucleic Acids Res.* **31**, 406–9 (2003).

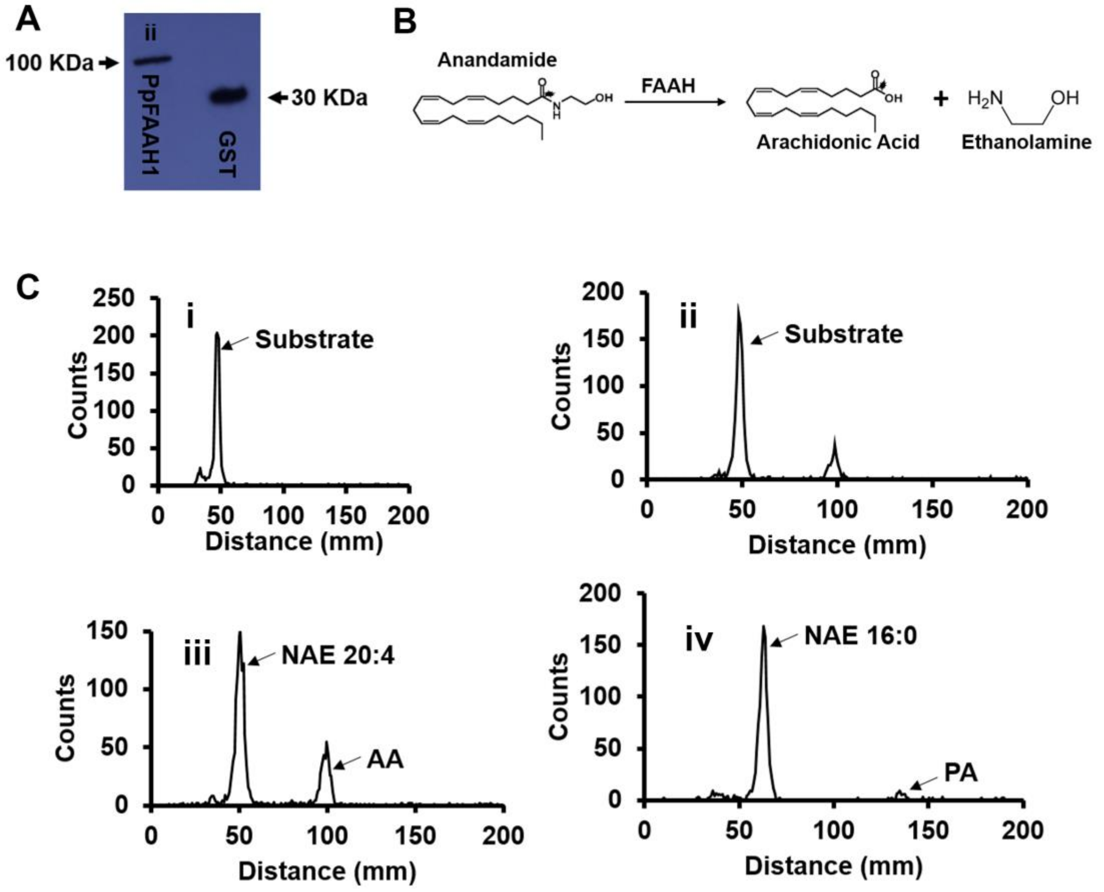

Fig. S1. Amide hydrolase activity and representation of radio-chromatograms. A). Western blot of purified GST-tagged PpFAAH1 (~100 KDa) and GST protein (~30 KDa) alone (as a control). B). Hydrolysis of anandamide by FAAH generates a free fatty acid, arachidonic acid and ethanolamine as products. The substrate is radiolabeled with $_{14}$[C] and upon hydrolysis, the radiolabel is retained by the free fatty acid product, allowing for quantification of FAAH activity. C). Representative chromatograms generated by the TLC bio-scanner. Substrate peaks are retained around 50 mm and product at ~100 to 150 mm from the point of origin on TLC plate. i) negative control with no enzyme and/or GST protein, ii) positive control using AtFAAH as the enzyme source, and iii) and iv) are with substrate NAE 20:4 and NAE 16:0 using PpFAAH1 as the enzyme source. Peaks AA (iii) and PA (iv) represent free arachidonic and palmitic acids as product of amidohydrolase activity of PpFAAH1.

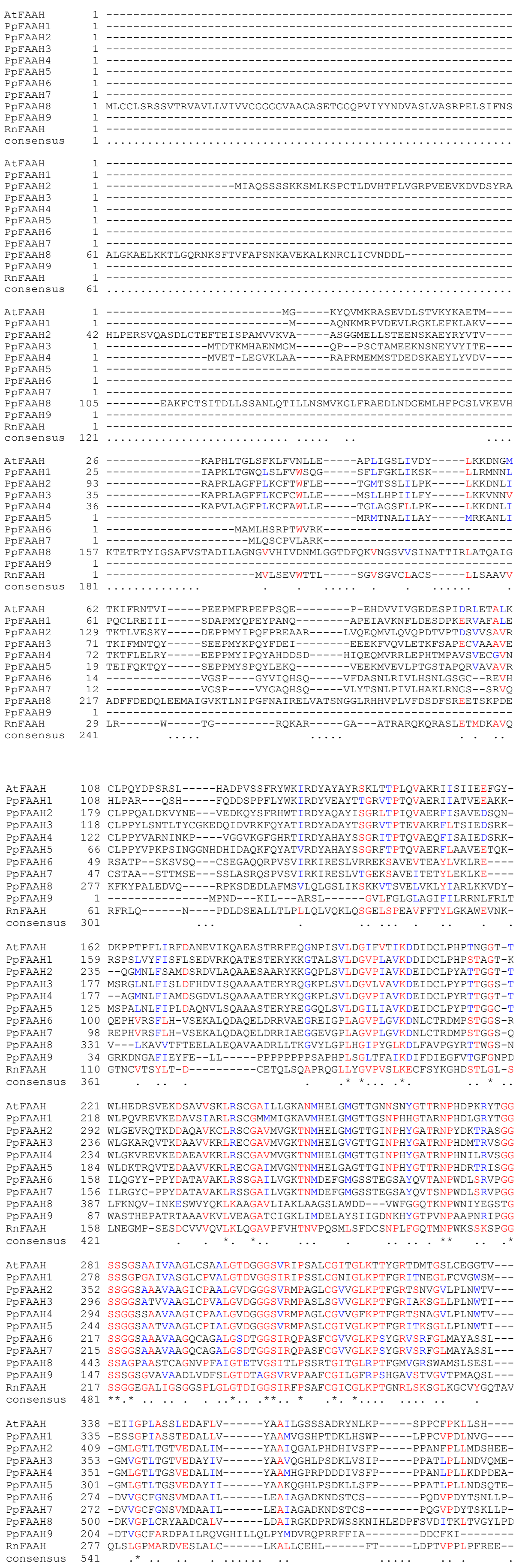

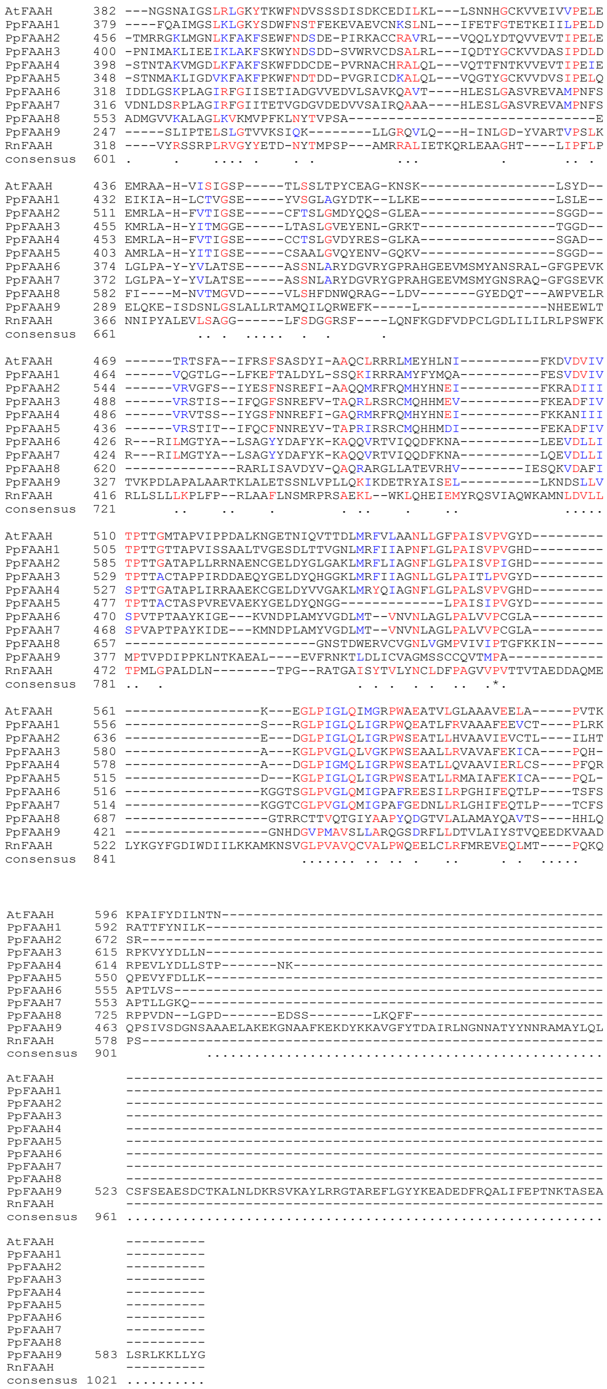

Fig. S2. Alignment of full-length sequences of nine PpFAAH paralogs with AtFAAH and RtFAAH. Shortened or extended N and C termini along with conserved amidase signature region among the proteins can be compared. The symbols: asterisk, dot and gap for the consensus sequence indicate identical, same class and different class of residues at the same position, respectively. Red, green, and black colors also represent the same order of consensus symbols in terms of conserved residues.

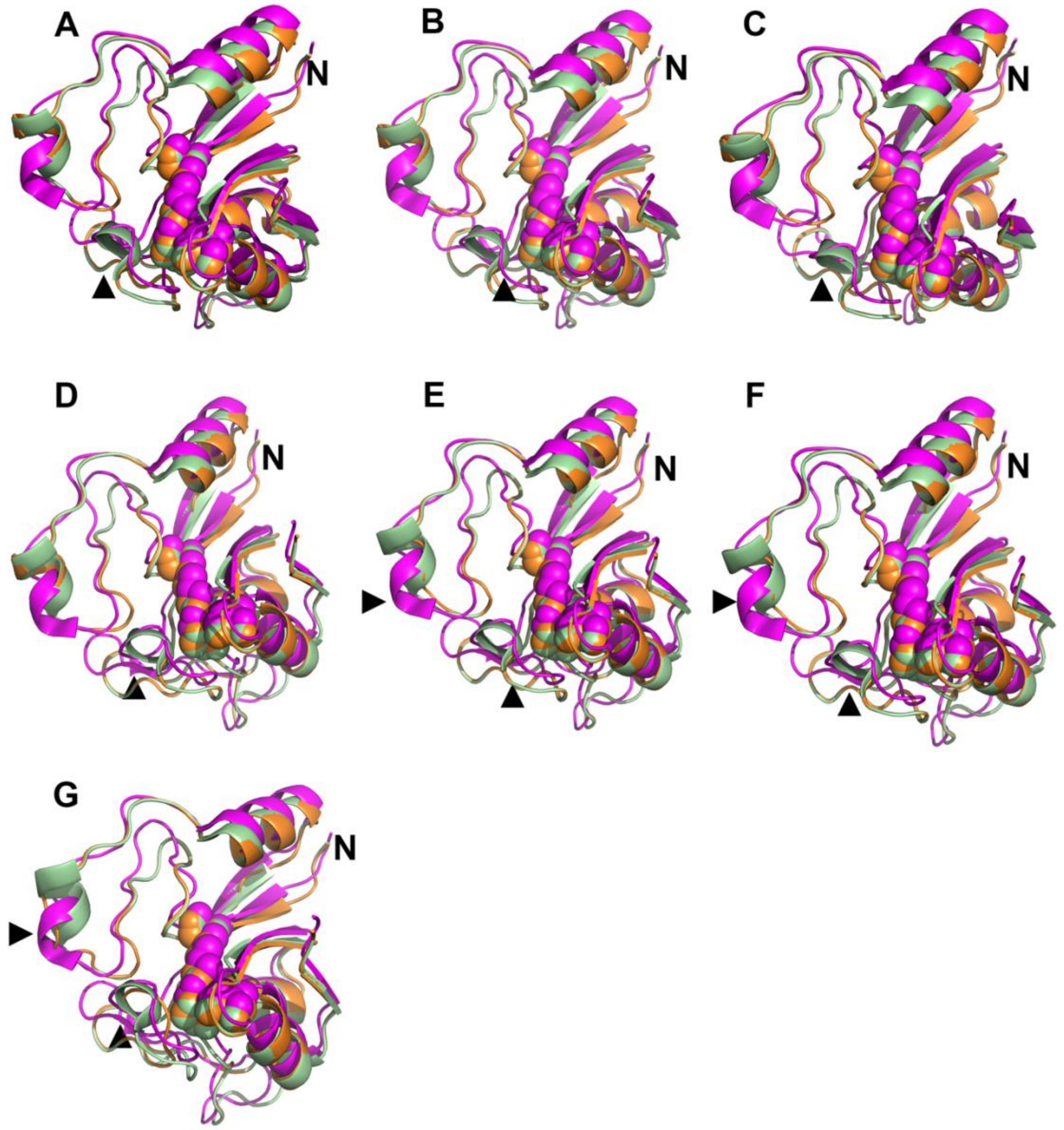

Fig. S3. Structural alignment of amidase signature (AS) region. Amidase signature region of AtFAAH (pale green), rFAAH (magenta) and PpFAAH (orange) were done using Chimera 5 software. A – G represents PpFAAH2 to PpFAAH8 aligned with both At and RtFAAH. Catalytic triad (Lys-Ser-Ser) are shown as sphere. N represents the N-terminus region of AS sequence. Arrows points to differences in alignment of helices and loops.

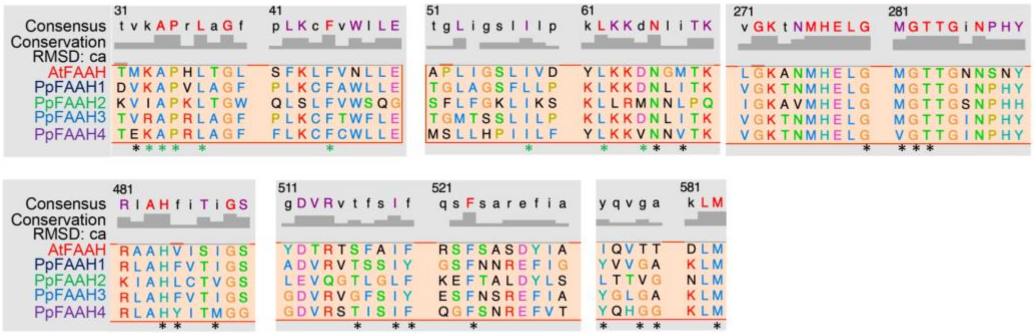

Fig. S4. Structural alignment of membrane binding cap (MBC) and membrane access channel (MAC). Predicted structures of PpFAAH1 to PpFAAH4 were aligned with AtFAAH to determine MBC and MAC of PpFAAH. Green and black asterisks at the bottom of the sequence represents the important residues that make the MBC and MAC, respectively. Shadow height shown above the sequences indicates the conservation of residues, whereas the numbers indicate the consensus alignment of the residues.

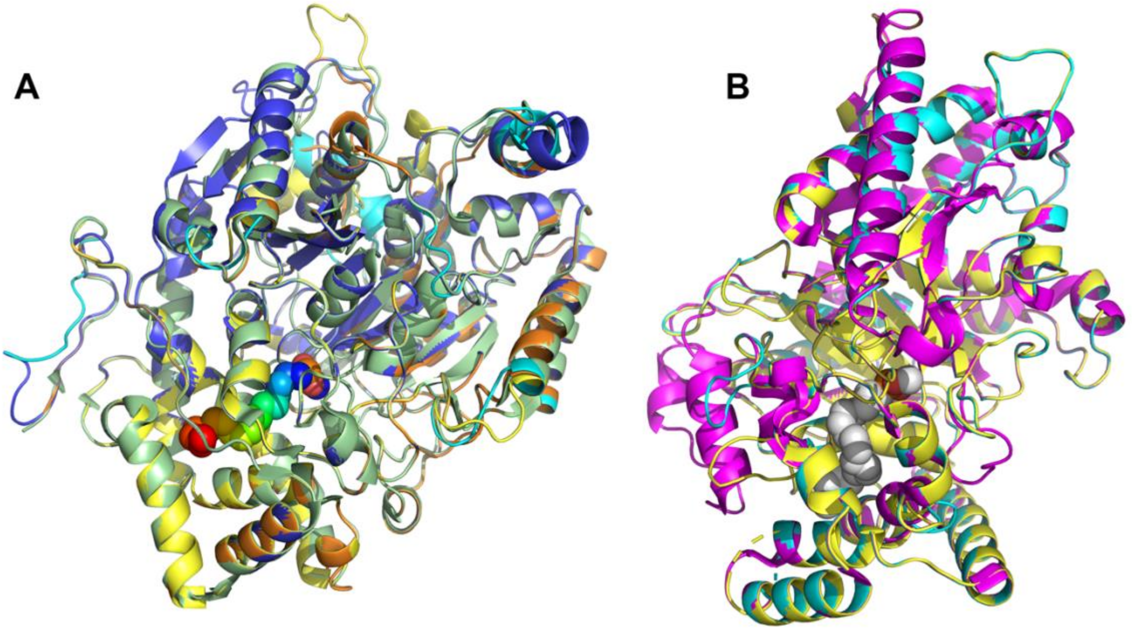

Fig. S5. Substrate docking of PpFAAH. A). Overlaid structural alignment of PpFAAH1 to PpFAAH4 with At FAAH1 as a template (pale green) and B). PpFAAH6 and PpFAAH7 with Rt substrate MAFP. Protein structures are shown as cartoon with the substrate as sphere.

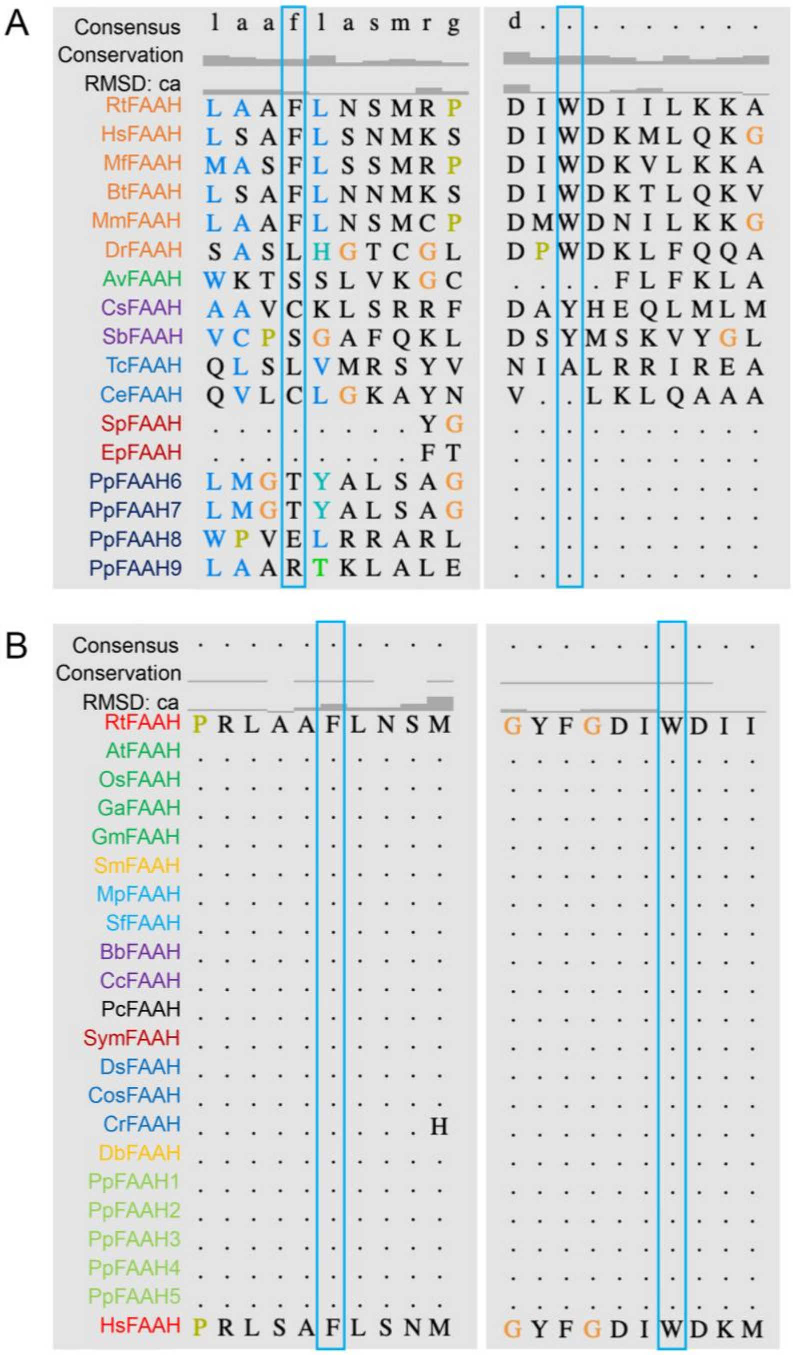

Fig. S6. Sequence alignment of dynamic paddle residues. Predicted structural sequence alignment of template RtFAAH (1mt5) with FAAH from Kingdom A). Animalia and PpFAAH6 to PpFAAH9, and B). Plantae, Fungi, Chromista, Protozoa and PpFAAH1 to PpFAAH5 using Chimera 5 to determine the potential dynamic paddle. Blue boxes in the alignment shown the residues that potentially can make the dynamic paddle. Shadow height shown above the sequences indicates the conservation of residues, whereas the numbers indicate the consensus alignment of the residues. For description of protein names, see Table S2.

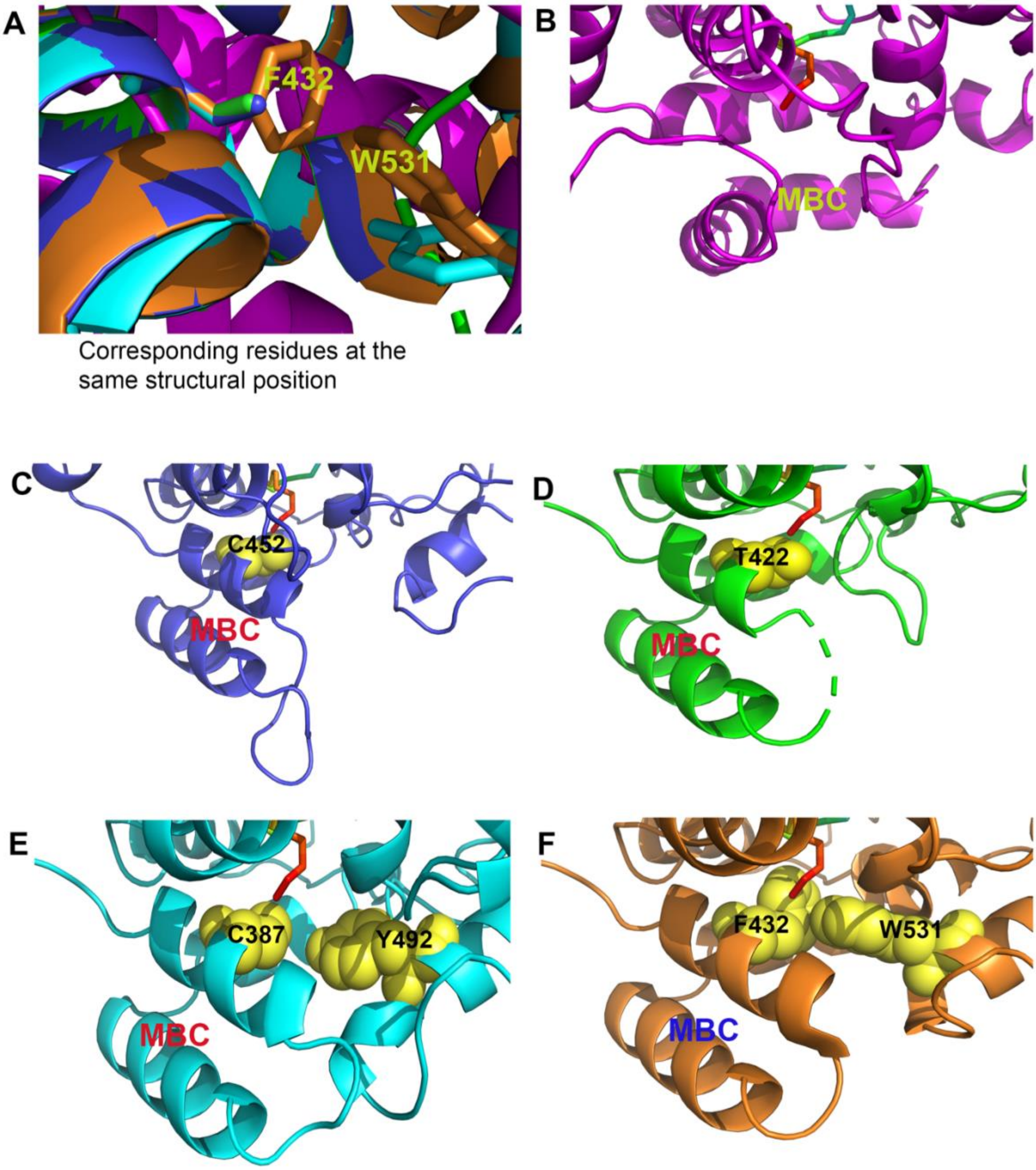

Fig. S7. Comparison of dynamic paddle region of FAAH. A). Overlay of secondary structures of dynamic paddle region with F432 and W531 residues of RtFAAH with EpFAAH, CeFAAH, CsFAAH and PpFAAH6. Corresponding residues for the orthologs at the position of F432 and W531 are indicated by red arrows; Close-up images of probable dynamic paddle region of B). EpFAAH, C). CeFAAH, D). PpFAAH6, E). CsFAAH, F). RtFAAH. Images B to E also show the substrate MAFP (sticks, rainbow in color), residues of dynamic paddle region (sphere, yellow in color), and the two α-helices that are predicted to make MBC.

Table S1. Details of PpFAAH paralog sequences along with AtFAAH, RtFAAH and HsFAAH

Name	Gene ID	Chromosome	Protein		Isoelectric point	Motif prediction (AA)	% Identity with		
			Length (AA)	Size (Kda)			AtFAAH	RtFAAH	HsFAAH1
AtFAAH	AT5G64440 (TAIR)	5	607	66.08	6.18	Amidase (198-321)	100	34	33
PpFAAH1	Pp3c23_9920V3.11	23	601	65.9	6.84	Amidase (195–318)	47	30	28
PpFAAH2	Pp3c3_2980V3.1	3	673	73.5	5.93	Amidase (269–392)	46	33	34
PpFAAH3	Pp3c7_18330V3.6	7	624	68.5	6.35	Amidase (213–336)	45	35	35
PpFAAH4	Pp3c26_13660V3.1	26	627	68.06	6.92	Amidase (211–334)	45	33	35
PpFAAH5	Pp3c11_92003.2	11	559	60.78	6.57	Amidase (161–284)	44	31	31
PpFAAH6	Pp3c27_1950V3.5	27	560	59.4	6.77	Amidase (135–257)	30	35	33
PpFAAH7	Pp3c16_16670V3.1	16	560	58.99	6.22	Amidase (133–255)	31	33	33
PpFAAH8	Pp3c4_17250V3.2	4	743	80.45	6.84	Amidase (364–483), Fascilin	26	28	28
PpFAAH9	Pp3c21_15890V3.1	21	592	64.35	7.96	Amidase (63–187), Tetratricopeptide repeat	39	32	32
RtFAAH	100911581 (NCBI)	5	579	63.35	8.49	Amidase (135-257)	34	100	82
HsFAAH	2166 (NCBI)	1	579	63.07	7.82	Amidase (135-257)	31	82	100

Table S2. Percent identity matrix for PpFAAH paralogs, along with AtFAAH and RtFAAH

%*	AtFAAH1	PpFAAH1	PpFAAH2	PpFAAH3	PpFAAH4	PpFAAH5	PpFAAH6	PpFAAH7	PpFAAH8	PpFAAH9	RtFAAH
AtFAAH1	100.0	46.4	44.4	44.3	45.0	45.3	26.3	26.9	24.5	22.6	22.5
PpFAAH1	46.4	100.0	43.5	44.2	43.7	46.3	29.6	29.8	21.8	21.4	24.3
PpFAAH2	44.4	43.5	100.0	59.5	56.6	71.6	27.7	26.9	22.4	23.9	22.2
PpFAAH3	44.3	44.2	59.5	100.0	71.8	61.1	28.0	28.2	19.2	23.8	22.0
PpFAAH4	45.0	43.7	56.6	71.8	100.0	58.6	28.2	28.7	20.3	23.8	21.7
PpFAAH5	45.3	46.3	71.6	61.1	58.6	100.0	28.9	29.5	22.0	23.3	23.5
PpFAAH6	26.3	29.6	27.7	28.0	28.2	28.9	100.0	85.6	23.0	20.6	24.7
PpFAAH7	26.9	29.8	26.9	28.2	28.7	29.5	85.6	100.0	22.7	22.0	23.9
PpFAAH8	24.5	21.8	22.4	19.2	20.3	22.0	23.0	22.7	100.0	20.1	21.5
PpFAAH9	22.6	21.4	23.9	23.8	23.8	23.3	20.6	22.0	20.1	100.0	18.3
RtFAAH	22.5	24.3	22.2	22.0	21.7	23.5	24.7	23.9	21.5	18.3	100.0

* Created by Clustal2.1

Table S3. Details of the organisms of which FAAH was used for phylogenetic and 'Dynamic Paddle' analyses

Kingdom	Phylum/Division	Scientific Name	Code	Protein ID*
Animalia	Chordata	*Rattus norvegicus*	RtFAAH	NP_077046.1
Animalia	Chordata	*Homo sapiens*	HsFAAH	NP_001432.2
Animalia	Chordata	*Marmota flaviventris*	MfFAAF	XP_027793438.1
Animalia	Chordata	*Bos Taurus*	BtFAAH	XP_024845397.1
Animalia	Chordata	*Mus musculus*	MmFAAH	NP_034303.3
Animalia	Chordata	*Danio rerio*	DrFAAH	NP_001103295.1
Animalia	Arthropoda	*Armadillidium vulgare*	AvFAAH	RXG52942.1
Animalia	Platyhelminthes	*Clonorchis sinensis*	CsFAAH	RJW72397.1
Animalia	Platyhelminthes	*Schistosoma bovis*	SbFAAH	RTG91481.1
Animalia	Nematoda	*Toxocara canis*	TcFAAH	KHN72390 .1
Animalia	Nematoda	*Caenorhabditis elegans*	CeFAAH	Q17449
Animalia	Cnidaria	*Stylophora pistillata*	SpFAAH	PFX22943.1
Animalia	Cnidaria	*Exaiptasia pallida*	EpFAAH	KXJ22769.1
Plantae	Angiosperms	*Arabidopsis thaliana*	AtFAAH	AT5G64440.1
Plantae	Angiosperms	*Oryza sativa*	OsFAAH	XP_015633439.1
Plantae	Angiosperms	*Gossypium arboreum*	GaFAAH	XP_012462809.1
Plantae	Angiosperms	*Glycine max*	GmFAAH	XP_003545751.1
Plantae	Lycopodiophyta	*Selaginella moellendorffii*	SmFAAH	EFJ12565.1
Plantae	Bryophyta	*Marchantia polymorpha*	MpFAAH	PTQ37667.1
Plantae	Bryophyta	*Sphagnum fallax*	SfFAAH	Sphfalx0000s0679.2
Fungi	Ascomycota	*Beauveria bassiana*	BbFAAH	PMB70458.1
Fungi	Ascomycota	*Cladophialophora carrionii*	CcFAAH	OCT49863.1
Chromista	Heterokontophyta	*Phytophthora cactorum*	PcFAAH	RAW37580.1
Chromista	Domain: Eukaryota	*Symbiodinium microadriaticum*	SymFAAH	OLP90858.1
Chromista	Algae, Chlorophyta	*Dunaliella salina*	DsFAAH	Dusal.0428s00008.1
Chromista	Algae, Chlorophyta	*Coccomyxa subellipsoidea*	CosFAAH	XP_005650878.1
Chromista	Algae, Chlorophyta	*Chlamydomonas reinhardtii*	CrFAAH	PNW86966.1
Protozoa	Amoebozoa	*Dictyostelium discoideum*	DdFAAH	XP_643382.1
Plantae	Angiosperms	*Arabidopsis thaliana*	AtAmidase	At1G08980.1
Animalia	Chordata	*Homo sapiens*	HsAmidase	NP_777572

*Protein sequences were obtained from either NCBI, Uniport or Phytozome 12 database

Table S4. Details of predicted secondary structure of PpFAAH orthologs with AtFAAH and RtFAAH as templates

Protein	Template 6DII (AtFAAH)							Template 1MT5 (RtFAAH)						
	**Aligned**	**Coverage (%)**	**Residues**	**# of α-helixes**	**# of β-sheets**	**Q score**	**RMSD**	**Aligned**	**Coverage (%)**	**Residues**	**# of α-helixes**	**# of β-sheets**	**Q score**	**RMSD**
AtFAAH1	605	100	(1-605)	23	17	0.99	0.27	464	74	(100-591)	20	11	0.67	0.65
PpFAAH1	596	99	(4-601)	23	10	0.94	0.54	433	72	(127-582)	18	11	0.70	0.50
PpFAAH2	573	90	(83-666)	25	8	0.90	0.42	436	65	(133-600)	18	11	0.71	0.51
PpFAAH3	603	96	(11-624)	23	10	0.94	0.46	447	72	(145-598)	16	13	0.70	0.46
PpFAAH4	596	95	(17-623)	24	8	0.92	0.46	435	69	(199-665)	19	11	0.70	0.46
PpFAAH5	548	98	(1-559)	21	8	0.85	0.45	444	79	(81-545)	18	10	0.70	0.62
PpFAAH6	516	92	(2-558)	22	9	0.77	0.62	477	85	(52-539)	21	11	0.83	0.45
PpFAAH7	482	86	(46-556)	18	8	0.73	0.73	447	80	(41-541)	22	11	0.83	0.47
PpFAAH8	425	57	(293-728)	16	8	0.62	0.68	426	57	(291-718)	18	11	0.70	0.61
PpFAAH9	442	75	(6-454)	17	8	0.62	0.83	434	73	(4-446)	16	11	0.75	0.56
RtFAAH	440	80	(78-578)	18	8	0.75	0.38	537	100	(38-573)	22	11	1.00	0.00

Table S5. Predicted dimerization residues of PpFAAH1 to PpFAAH4, relative to AtFAAH

**AtFAAH***	**PpFAAH1**	**PpFAAH2**	**PpFAAH3**	**PpFAAH4**
Gln5	Asn4	-	Pro15	-
Arg66	Arg65	Val133	Met75	Leu76
Thr68	Ile67	Ser135	Thr77	Leu78
Phe76	Tyr75	Tyr143	Tyr85	Tyr86
Asp225	Val222	Val296	Ala240	Val238
Thr454	Ala450	Gly529	Gly473	Gly471
Pro455	Gly451	Met530	Val474	Val472
Phe479	Phe474	Phe554	Phe498	Phe496
Ala481	Ala476	Ser556	Asn500	Asn498

*Substitution by same (green) or different class (red) of residue relative to AtFAAH

Table S6. Summary of predicted model for membrane binding cap

Protein	Terminus (AA position)	# of hydrophobic residues	# of helixes	Predicted model
AtFAAH1	N (27-60)	21/34	α1 and α2	Teeth on a comb
PpFAAH1	N (L21-P61)	24/41	"	"
PpFAAH2	N (A94-L127)	19/37	"	"
PpFAAH3	N (L39-L61)	19/23	"	TM and membrane integrated
PpFAAH4	N (A37-I71)	24/31	"	"
PpFAAH5		*Not available*		
PpFAAH6	C (A413-L435)	14/23	α18 and α19	Teeth on a comb
PpFAAH7	C (V422-F441)	14/20	"	"
PpFAAH8	C (A602-V616)	-	α12	"
PpFAAH9	C (V258-L271)	-	α9	"
RtFAAH	C (404-433)	23/34	α18 and α19	"

Table S7. Comparison of the residues at the entrance of ligand binding pocket

AtFAAH1*	PpFAAH1	PpFAAH2	PpFAAH3	PpFAAH4
Ala27	Ala26	Ala94	Ala36	Ala37
Pro28	Pro27	Pro95	Pro37	Pro38
Leu30	Leu29	Leu97	Leu38	Leu40
Phe38	Phe37	Phe108	Phe47	Phe48
Ile51	Ile50	Ile118	Ile60	Leu61
Leu55	Leu45	Leu122	Leu64	Leu65
Lys26	Ile25	Arg93	Lys35	Lys36
Asp58	Met57	Asp125	Val67	Asp68

*Substitution by same (green) or different class (red) of residue relative to AtFAAH

Table S8. Residues of substrate binding pockets in PpFAAH, relative to AtFAAH

AtFAAH1*	PpFAAH1	PpFAAH2	PpFAAH3	PpFAAH4	PpFAAH5
M25	V24	V92	E34	V35	-
A27	A26	A94	A36	A37	-
L55	L54	L122	L64	L65	M12
N59	N58	N126	N68	N69	N16
M61	L60	I126	V70	I71	I18
K205	K202	K276	K220	K218	K168
G255	G252	G326	G270	G268	G218
M256	M253	M327	V271	M269	A219
G257	G254	G328	G272	G270	G220
T258	T255	T329	T273	T271	T221
S281	S278	S352	S296	S294	S244
S305	S302	S376	S320	S318	S268
H441	H437	H516	H460	H458	H408
V442	L438	F517	Y461	F459	-
I445	V441	I520	M464	I462	I412
S472	T467	G547	T491	T489	T439
I475	L470	I550	I494	I492	-
F476	F471	Y554	F495	Y493	F443
F479	F474	F554	F495	F496	F446
I532	L527	Y607	Y551	Y549	-
T535	V530	G610	G554	G552	-
T536	G531	A661	G555	A553	-
M539	M534	M624	M558	M556	K493

*Substitution by same (green) or different class (red) of residue relative to AtFAAH

Table S9. Residues of substrate binding pockets in PpFAAH, relative to RtFAAH

RtFAAH*	PpFAAH6	PpFAAH7	PpFAAH8	PpFAAH9
K142	K140	K142	K371	K70
M191	G189	G191	A420	A121
L192	M190	M192	W421	Y122
S193	G191	G193	-	S123
F194	S192	S194	-	I124
G216	G214	G216	G442	G146
S217	S215	S217	S443	S147
T236	S234	S236	T462	T166
D237	D235	D237	E462	D167
I238	T236	T238	T464	T168
G239	G237	G239	V465	A169
G240	G238	G240	G466	G170
S241	S239	S241	S467	S171
Y335	G337	A339	-	Q269
L372	L374	L376	A580	R306
E373	P375	P377	E581	T307
S376	Y378	Y380	M584	Q310
A377	-	V381	N585	I311
L380	A381	-	M588	-
F381	T382	Y491	G589	-
L404	E405	E407	-	-
R428	-	-	A631	A335
A431	G429	G431	V616	A338
F432	T430	T432	E617	R339
T488	Y489	Y491	-	N399
G489	V490	V492	-	K400
I491	D492	D494	W662	L402
V495	V496	V498	C666	C406
W531	-	-	-	-

*Substitution by same (green) or different class (red) of residue relative to AtFAAH
